# Mechanisms linking individual and organizational culture change through action research: Creating change agents for organizational and food safety culture development

**DOI:** 10.1016/j.heliyon.2023.e13071

**Published:** 2023-01-19

**Authors:** Anne-Mette Olsen, Anna Marie Møller, Sanne Lehmann, Anders Vind Kiethon

**Affiliations:** a Department for Food Safety and Veterinary Issues, Danish Agriculture & Food Council, Axeltorv 3, 1609, Copenhagen V, Denmark; b Department of Psychology (Bachelor Student), University of North Georgia, 82 College Circle, Dahlonega, GA, 30597, USA; c Department of Disaster and Risk Management, Copenhagen University College, Humletorvet 3, 1799, Copenhagen V, Denmark; d MyElite Relationship-therapy (MER), Peter Bangs Vej 1, 2000, Frederiksberg, Denmark

**Keywords:** Food safety culture, Organizational culture, Change agents, Social capital, Underlying mechanisms, Therapeutic training, Senses and emotions, Training human competencies, Action research, Individual inquiry practice

## Abstract

This article proposes a more individualized approach to organizational and food safety culture development through the creation of culture change agents. The study used action research with individual therapeutic training of sensory and emotional skills as the action intervention to reveal underlying mechanisms of the culture and create long-term culture change. The study was conducted with a group of voluntary employees over a 3-year period at a department under Food Safety and Veterinary Issues in Danish Agriculture and Food Council. Data was collected using individual in-depth qualitative interviews with a novel questionnaire technique that facilitated participants to bring otherwise unconscious underlying assumptions to awareness. The study found that working intensely and therapeutically on an individual and group level, had a significant impact on the surrounding culture and social capital. Five underlying mechanisms were revealed linking individual culture change to changes in the social capital and culture of the department. Going through the five underlying mechanisms may enable individuals to get to the root causes of issues, facilitate more sharing and collaboration to learn from near-misses and failures, and take action despite facing uncomfortable situations, all important abilities to develop FSC. Based on the underlying mechanisms a ‘Change Agent Model’ was developed. The model illustrates the important underlying mechanisms that any individual or group can work through to become culture change agents and drivers for organizational culture and FSC development. This is the first of two articles.

## Highlights

• Action research was used as both overall design and individual inquiry practice.
• Employees engaged in therapeutic training to strengthen sensory and emotional skills.
• Underlying mechanisms of culture were revealed by inquiring from senses and emotions.
• Development in employee's internal state improved social capital and surrounding cultures.
• A change agent model for organizational and food safety culture development is presented.

## 1 Introduction

### 1.1 A need for FSC change agents to develop FSC

According to Global Food Safety Initiative (GFSI) and scholars, we need to combine traditional control and management strategies with practices that continuously develop an organization's *Food Safety Cultur*e (FSC), if we want to fully address present food safety challenges [[1], [2], [3]]. Developing a strong FSC is especially crucial to increase employees' ability to take meaningful action in critical situations and during unforeseen hazards, where following routine procedures are not an option [4]. Several international GFSI-approved private standards have adopted FSC as an integrated part of the standard for Food Safety to make regulations reflect desired practices [[5], [6], [7]]. However, it remains a challenge to change FSC in practice, as it encompasses both the shared values, norms, and beliefs of an institution. FSC affect Food Safety Operations on all levels of an organization and involve anyone directly handling or indirectly working with food and food production. FSC is continuously changing with the people of the organization, as it affects and is affected by employee's mindset, awareness of, and behavior towards food safety [1,8,9]. A study in 2017 revealed significant disparities between written procedures, and what was practiced in several food companies, illustrating the organizational FSC challenge of ‘walking the talk’ [10]. Despite this, previous assessment tools and food safety training has struggled to change employees' behavior long-term [[11], [12], [13], [14], [15]]. Through the use of action research, only one study has demonstrated a more long-term behavior change [16]. We need to train more FSC ambassadors, who are committed to long-term behavior change and can lead the culture transformation through action, as one study already highlights [17]. FSC ambassadors or change agents may be described as people who develop the cultures around them by continuously developing themselves, changing the way they interact with themselves and surroundings to such a degree that it facilitates transformation of the organizational culture and FSC. It has been strongly recommended to develop the people to improve FSC [18], and only by developing the people, can we develop the culture.

### 1.2 A strong FSC is a strong organizational culture

A strong FSC is characterized by a common purpose, trustful environment, strong involvement, shared responsibility, high information exchange, and strong relationships among employees and leaders [4,19]. These characteristics resemble an organization with high social capital and strong organizational culture, as social capital reflects the levels of trust, norms, and networks that facilitate collaboration and cooperation within the organization [20,21]. FSC is deeply connected with the organizational culture and its social capital, being part of “the way we do things around here” [4,22]. In practice, FSC and organizational culture cannot be separated, and both must be developed to build a strong FSC. Despite this, a literature review of FSC research between 2010 and 2021 found that existing research has primarily focused on the observable aspects of FSC without addressing underlying mechanisms and root causes that are often interchangeable with the organizational culture [18]. The study directly suggests a need to explore the underlying mechanisms of FSC through action research, to better understand FSC development in practice and improve food safety practices [18].

### 1.3 Training individuals to develop FSC and organizational culture

This article goes beyond the conventional approach to FSC and organizational culture development by proposing a more individualized approach using action research and training of human competencies. The study sought to gain practical insights into the experiential learning process of accessing and changing underlying mechanisms of any culture, including FSC and organizational culture, through the training of individual human competencies. The objective of the study was to expose and change deeper underlying mechanisms embedded in the culture of several individuals to build a stronger social capital and culture in the organization through the development of culture change agents. In the following section, culture as the theoretical framework is presented, followed by the methodological design focusing on therapeutic training as an action intervention capable of transforming culture.

## 2 Theoretical background

### 2.1 Underlying mechanisms: the unconscious essence of culture

Schein's theory was chosen since it is the most common and applicable understanding of culture used within FSC research and culture development in general [10,18,[22], [23], [24]]. Culture, according to Schein, can be described as a multilayered system made up of three layers: artifacts, belief systems, and underlying assumptions, see left part of Model 1. Artifacts represent the observable aspects of culture including actions, quantifiable results, and organizational structures. If culture is only addressed on an observable level, it seldomly creates long-lasting culture change, as the underlying assumptions of the culture challenges are left unaddressed [25]. Belief systems can be both conscious and unconscious, and include strategies, behavioral norms, and mindsets. Underlying assumptions are the unconscious taken-for-granted beliefs, perceptions, feelings, and thoughts, and is the most important layer of a culture to address for long-term change [25]. Underlying assumptions are the essence of any culture, including FSC, and the root cause from which any behavior or norm originate. Working with underlying assumptions often require an individualized approach involving therapeutic and emotional training to bring the unconscious to awareness [25]. Furthermore, when dealing with underlying assumptions in practice, there is no difference between working with FSC, organizational culture or any other culture, as they all belong to the same cultural essence within an individual. This is because the different contexts an individual engages with such as the workplace, family, friends etc. are deeply interconnected and intertwined on the unconscious level, see right part of Model 1.

**Model 1:**
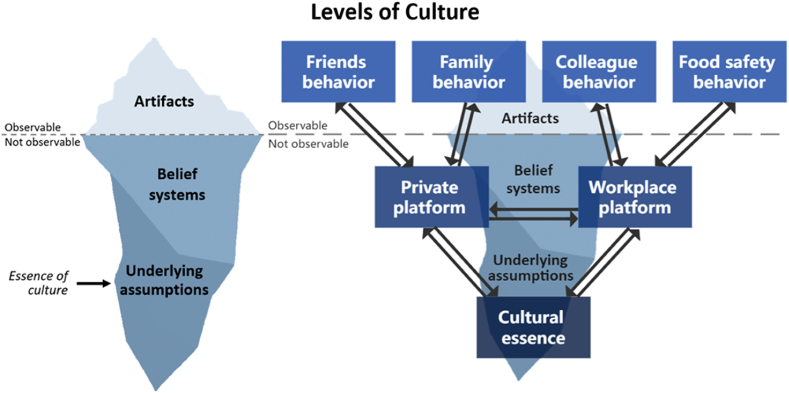
**Levels of Culture:** To the left: Illustration of Schein's different levels of culture with the iceberg illustrating what part of culture is observable and not observable. To the right: Illustration of Schein's different levels of culture as they relate to different cultural platforms and behaviors that an individual engages with. The model illustrates that when an individual changes their cultural essence (underlying assumptions), it can be expected to influence all cultures that the individual take part in.

### 2.2 Bringing underlying mechanisms to awareness

According to Schein, changing underlying assumptions may be described as a therapeutic process through which individuals resurrect, reexamine, and transform their internal states [25]. Using therapeutic training as an intervention to transform culture may foster more psychological safety to support individuals through the resurrection process despite anxiety and individual barriers [25–27]. A Danish therapeutic concept, MyElite Relationship-therapy (MER), founded by Anders Vind Kiethon, was used in this study as part of the theoretical framework and methodological approach and intervention. MER's therapeutic approach is based in neurophysiology research, which have shown that all human learning and development starts with sensing [28]. Feelings and thoughts cannot arise in the brain without there being a nerve signal first [29]. Any human development is inherently experientially based, learning by doing, as the ability to sense is the foundation of all input and exchange with surroundings [28]. According to MER, individuals have three fundamental human competencies to experience, engage with, and make sense of the world: sensing through the body, feeling various emotions as a reaction to sensations, and thinking/rationale comprehension based on the sensory experiences and emotional reactions, see Model 2.

**Model 2:**
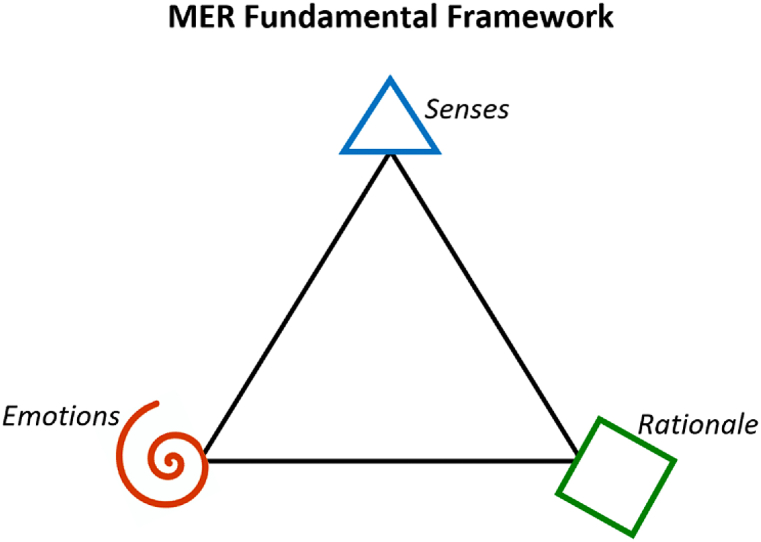
**MER Fundamental Framework:** According to MyElite Relationship-therapy (MER) humans fundamentally have three human competencies: sensing through the body (blue triangle); feeling various emotions as a reaction to sensations (red spiral); and thinking/rationale comprehension based on sensing and emotions (green square).

By connecting the therapeutic intervention (MER) and Schein's levels of culture model, the human competencies that need to be trained to address underlying assumptions of culture may be revealed. The rationale reflects Schein's artifacts, as they represent the most understandable and observable aspects of humans and culture, including FSC. When individuals work with their thoughts and rationale, they are usually addressing the artifacts (observable) layer of the culture and are unable to access the deeper layers as they are inherently unconscious and impossible to rationally decipher, see left side of Model 3. Senses and emotions collectively reflect the underlying assumptions and belief systems as they are an integrated part of the individual's unconscious taken-for-granted perceptions and emotional reactions. Working with senses and emotions may therefore allow individuals to recognize and reshape underlying assumptions and belief systems by actively becoming aware of and engaging with one's internal state, see right side of Model 3. This may consequently, cause changes in thoughts and behavior patterns (artifacts), including FSC related behaviors, as the artifacts can always be expected to change when underlying assumptions change [25].

**Model 3:**
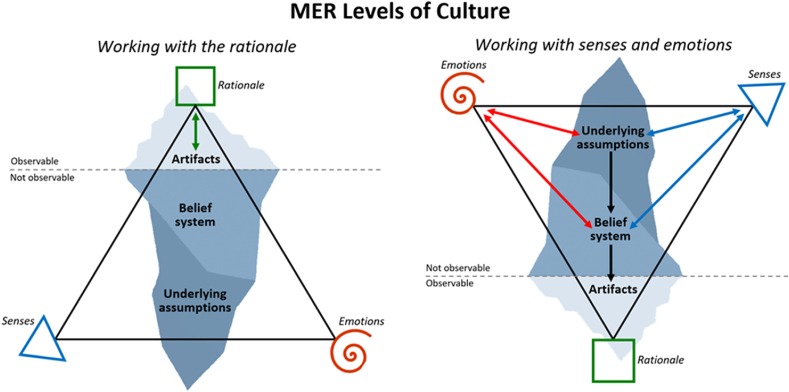
**MER Levels of Culture:** Illustration of Schein's levels of culture and MER fundamental framework together, illustrating that working with senses and emotions (as opposed to just the rationale) may enable individuals to gain access to and transform underlying assumptions.

## 3 Materials & method

### 3.1 Action research as study design and individual inquiry practice

The aim of action research is to create changes in practice and reveal underlying mechanisms through repeated circles of planning, action intervention, and reflection [30]. The action intervention reveals underlying mechanisms by disturbing frozen structures to make them unfrozen so they can be addressed, and positive change can be accomplished [31]. The underlying mechanisms resemble underlying assumptions in Schein's culture model, as they are hidden beliefs, perceptions, and mindsets from which behavior patterns originate [31]. Addressing frozen structures through an action intervention allows an organization or group to change from frozen (old status quo) to unfrozen (transformative stage) to a better refrozen stage (new status quo) [30]. During the unfrozen transformative stage participants may gain access to underlying assumptions of the previous frozen structures, making it possible to change and establish new and better refrozen structures as illustrated in the left part of Model 4. This study used MER therapeutic training as the action intervention. The training involved working with sensory and emotional skills, which requires an explorative study method that allows for deeper complex and unknown mechanisms to be revealed [28,32,33]. Action research was used as the explorative method as it allows for unpredictable changes during the study to be reflected upon and implemented [31]. The MER therapeutic training was centered around learning and mastering the MER Inquiry Practice to increase participants' ability to purposefully access their underlying assumptions and transform their internal states. To do so a shared language of words, symbols, and colors were taught to better distinguish senses, emotions, and rationale. The MER Inquiry Practice resembles action research's three stages of frozen-unfrozen-refrozen on an individual level, where identifying a specific context, focusing on your senses, and taking deep breaths act as the action intervention. As illustrated in the right side of Model 4, an individual thereafter asks themselves the same three questions in the same order no matter the context:

1. Unfrozen stage: When focusing on the context, what do I sense in my body?
2. Unfrozen stage: Having these senses, how does it feel emotionally?
3. Refrozen stage: How can it be that I sense and feel this way in this specific context?

**Model 4:**
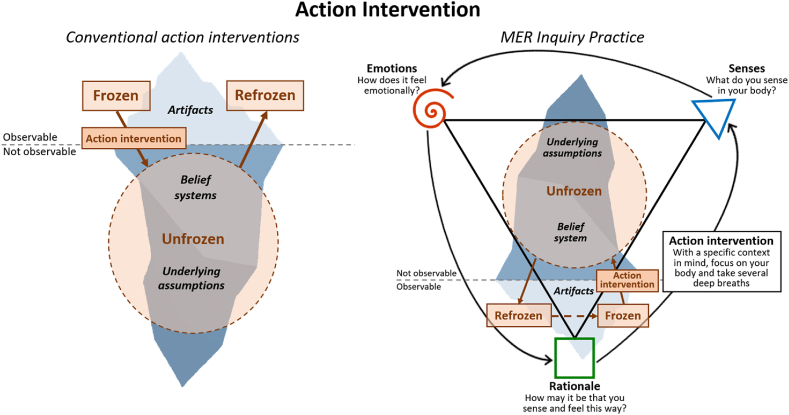
**Action Intervention**: To the left: Illustration of going from frozen to unfrozen to refrozen when transforming culture. To the right: Illustration of MER Inquiry Practice which uses a specific context, focusing on one's senses, and deep breaths as the action intervention to reveal underlying assumptions. Through therapeutic training, participants were taught to perceive and distinguish senses and emotions (unfrozen stage) and create meaning based on them (refrozen stage). Refrozen structures may be continuously addressed by repeating the MER Inquiry Practice.

The contexts included personal cases from the workplace and other relational platforms that participants identified as important to improve their individual culture. When going through the MER Inquiry Practice, participants were therapeutically guided to distinguish their sensations and emotions, and create rationale meaning, connecting what they sensed and felt with the specific context. Participants were therapeutically guided to be curious into any emotional resistance that occurred in them, making it a new context focus so they could slowly get to the root cause of their own individual culture. Based on the meaning arrived from their sensory and emotional experiences, participants revealed underlying assumptions of their culture and could thereby choose to change behavior, arriving at refrozen stage. Refrozen structures may be continuously addressed by repeating the MER Inquiry Practice, continuously making the refrozen the new frozen to be addressed. Action research was therefore both used as overall study design (left part of Model 4) and reflects the individual inquiry practice that all participants were trained in during the action intervention (right part of Model 4).

### 3.2 Case study in researchers’ own organization

Action research was used in the researcher's own organization. This is common in action research where the researcher takes a more active role to initiate and facilitate the change process. Studying one's own organization allows the researcher to have more practical experiences and insights into the challenges, the organization is facing, and helps to ensure the learned continues to be applied in the organization to similar challenges in the future [[34], [35], [36]]. This study worked with a specific case to allow for a deeper understanding of underlying mechanisms and higher transferability to practice by considering contextual and individual elements and barriers [37]. The case took place in a department under Food Safety and Veterinary Issues in Danish Agriculture & Food Council during a 3-year period (n = 11). The department has a strong professional and technical foundation, working with legislation, codes, and standards relevant to safe food production and FSC including food safety audits and inspections. The department also works in close contact with food companies, national and international legislators, and international organizations to address practical implementations of food safety and FSC development. The department has traditionally worked individually and in closed off sectors, but the nature of the department's work is changing partly due to the digital and technological development. Work tasks have changed involving more complex and interdisciplinary challenges that have no simple solutions, demanding increased collaboration and stronger relationships across sectors and stakeholders. In addition, a generational shift is on the way, creating an urgent need to increase collaboration and experience sharing to prepare onboarding employees for the retirement of older generations, and ensure no bigger projects are put solely on one person's shoulders. The department wanted to experience a culture transformation themselves to both develop their culture and get practical insights into the development of FSC, as the department plays a significant role in advising others on best FSC practices and improvements. Voluntary employees went through intensive therapeutic training with MER to increase their sensory and emotional skills with the purpose of learning to continuously change their underlying mechanisms and thereby influence the culture and social capital of the department.

### 3.3 Action research circles and structural development of study

The study consisted of three general action research circles. During study circle 1, the formation of a steering group to facilitate the culture development process was created and voluntary 2–3 hours individual and group MER sessions were offered to the employees in the department on a biweekly to monthly basis. Five voluntary employees started individual sessions, some continued in group sessions and two employees stopped again. The steering group wanted to increase ownership among the voluntary employees to strengthen the collective effort toward culture development in the department. So, after 13 months, the steering group decided to merge the two groups into one focus group marking the beginning of study circle 2. In the focus group participants engaged as co-researchers, allowing for a more democratic process in addressing the culture development of the department [30,31,38]. The focus group wanted to further engage all employees in the culture development process and therefore planned and executed two department-wide workshops across a seven-month period. All three voluntary employees continued individual sessions and participated in the focus group, and an additional voluntary employee started individual sessions. The focus group experienced emotional resistance to culture change from employees not engaging in individual sessions during both workshops and at the workplace in general. The focus group therefore decided to pause workshops and instead intensify the focus group work to become drivers of the culture change themselves, marking the beginning of study circle 3. All 4 employees continued doing individual sessions and focus group sessions were doubled. A cross-sectional FSC group was also created to facilitate collaboration, increase experience sharing, and coordinate activities and counseling efforts related to FSC development. After a seven-month period the study ended. The cross-sectional FSC group and focus group continued their work with culture development.

### 3.4 Interview questionnaire technique using MER Inquiry Practice

The study used individual in-depth qualitative interviews as its primary source of data collection. The interviews were designed to reveal the participants' process of transforming their underlying assumptions by going through the MER Inquiry Practice with each question, see Example Question, Table 1. This allowed participants to gain access to their sensory and emotional responses to each question before answering rationally. Participants were therefore not interviewed until later in the study to allow them to train and fully familiarize themselves with the MER Inquiry Practice first. The interviews included open-ended questions concerning employee's experience with MER, general effects of the training on both personal and work life, and questions addressing the social capital at the workplace. The social capital questions were based on the guideline developed by the Danish Working Environment Council with additional exploratory questions as defined by Steinar Kvale [39,40]. Data was collected during study circle 2 and 3 of the study. Six in-depth individual interviews (2 hours each) were conducted among four employees. Two employees were interviewed in circle 2, and all four employees were interviewed in circle 3 of the study. After the first round of interviews in circle 2, small updates were made to the interview guide for circle 3 interviews, changing the order of some questions for clarity and adding an in-depth guided MER Inquiry Practice for participants to explore what was needed for them personally to drive the culture development. List of interview questions may be shared upon request. Summaries and posters were collected from all group sessions, focus group meetings, and departmental workshops, including questionnaires from before and during the workshops (n = 11). The questions during the workshops addressed workplace-related cases and social capital using the MER Inquiry Practice.

**Table 1:** **Example Question**: Question from individual interview guide.

**Can employees express their opinions and feelings?** Breathe deeply and focus on the question. *Senses:* What do you sense in your body? *Emotions:* Having these senses, how does it feel emotionally? *Rationale:* How can it be that you sense and feel this way in regard to this question?

The study followed the Danish ethical requirements for social science studies as laid out by the Danish National Center for Ethics. The study used informed consent, confidentiality, transparency, and validation of quotes, which are recommended ethical practices when doing qualitative interviews [40]. All aspects of the study, including the individual MER sessions, group formations, interviews, and workshops, were voluntary, and participants could at any time leave the study with no repercussions. Participants were informed about the depth and purpose of the therapeutic training throughout the whole study. The data was analyzed by two researchers to minimize any potential bias. The researchers simultaneously engaged in continuous discussion of the relationships between experiences of change, underlying mechanisms, and change processes to ensure credibility. All data was anonymized.

### 3.5 Analyzing for changes in individual culture and surroundings

Interviews were transcribed and qualitatively analyzed according to the methods for meaning coding described by Steinar Kvale and Svend Brinkmann [40]. Repeated detailed readings of raw data were performed to identify experiences of change that indicated transformation in participants' internal states (individual culture) and social relationships/capital (organizational culture). Experiences of change were then extracted from the data and grouped into underlying mechanisms based on what was changed and the consequence of it. Underlying mechanisms were further grouped into change processes to illustrate the ways in which transformation of participants' internal states unfolded. Three different change processes were identified during the study: tipping point, step-by-step, and mastery, see Table 2. Tipping points reflected a significant transformation taking place within the internal state of participants. Step-by-step was defined as incremental change to one's internal state over a longer period of time. Lastly, mastery meant actively engaging to develop one's internal state further. The data collected throughout the two workshops had substantially shorter responses and showed no clear underlying mechanisms. Posters collected throughout the focus group and cross-sectional FSC group was used to help define concepts developed during the study period.

**Table 2:** **Change Processes:** Change processes illustrate the ways in which transformation of the participants’ internal states unfolded. Three change processes were identified during the study: tipping point, step-by-step, and mastery.

Change processes	Description
Tipping point	A significant transformation taking place within one's internal state
Step by step	Incremental change to one's internal state over a longer period
Mastery	Actively engaging to develop one's internal state further

## 4 Results

The four participants who were interviewed represented both genders (male and female), onboarding and experienced employees, veterinarians and other educational backgrounds, and an average age of app. 50 y/o. The study identified 5 underlying mechanisms that participants went through as they trained and engaged to actively change their own individual culture and the culture of the department: accepting senses as the internal navigator, embracing one's emotions unconditionally, witnessing oneself and surroundings, interacting openly with others, and practicing Safe Harbor. All underlying mechanisms were present in all interviews. In the following section, each underlying mechanism is described in-depth followed by a model illustrating all 5 underlying mechanisms together and their relationships to each other.

### 4.1 Learning to accept senses as internal navigator

Before initiating therapeutic training, participants had been accustomed to navigating their lives through the rationale, especially in work settings where the primary focus was often on technical and rational skills and performance. It was therefore an important tipping point for participants to let go of rational control and accept senses as the internal navigator. Learning to sense their body and experiencing the value of it was time-consuming and took a lot of training, primarily due to an unfamiliarity with using senses actively in the workplace. The process therefore at times generated feelings of frustration and impatience, as participants were used to succeeding at something as soon as they understood it, which wasn't possible in this case, as one participant expressed:

*'The impatience definitely arises because you want to be an expert right away, however that is not possible with this, because you have to walk the road and that demands a lot of internal persuasion, before you completely accept that that is actually how it works. You cannot understand your way to it, and you may listen and understand that you can’t understand your way to it, but from there and actually arriving at acceptance, there is some way to go.’*

Furthermore, rational control often provided a false sense of insurance in being right, as participants were constantly analyzing the situation to try and live up to their ideals and outside expectations. Having to let go of rational control to become present therefore took a lot of courage, as one participant expressed:

*‘I have [previously] suppressed all my emotions with my intellect, so for me to finally surrender and let go of my ‘I want to be right’ that was actually really transformative, because I dared to let go and I did not lose myself, but that was what I had imagined would happen. That was really interesting.'*

As participants accepted senses as the navigator, several expressed feelings of liberation and joy in finally being able to put into play aspects of themselves that had previously been suppressed.

### 4.2 Learning to embrace one's emotions unconditionally

The emotional tipping point took place when participants were able to embrace their emotions unconditionally as an integrated part of themselves including both negative and positive emotions. This was a transformative experience, as participants had previously suppressed their emotions and tried to distance themselves from uncomfortable emotions. As participants began to access their emotions through the senses as opposed to the rationale and slowly allowed more emotions to exist freely within themselves, they found even the negative and uncomfortable emotions to be useful, as one participant said: ‘*I experience both the positive and negative [feelings] as being true, as in positive even if they hurt’.* Participants found that if they wanted to create a more collaborative and trustful culture, they first had to trust and embrace all aspects of themselves, as one participant said:

*‘The feeling of being in balance with oneself, which helps me be better to myself, and that becomes a new ripple in the water with others, and wanting them, and wanting to build these relationships. So, it is like a flow, and what started out as a landmark for me, was the experience of having to give myself the big hug, the understanding that the parts of me that I was critical about or didn’t believe was good enough, really accept and give them love, that changed something, that was the button.'*

The more participants accepted themselves and their emotions, the better relationships, trust, and social capital they experienced with others. Furthermore, participants expressed that the focus group was an essential support, as they were able to share more emotional and vulnerable sides of themselves at the workplace, as one participant said: *‘The fact that I have felt an acceptance from all of you has helped me in accepting myself’.* As participants reached the tipping point and embraced their emotions unconditionally, they experienced having more to offer at the workplace, strengthening their personal capital and value as an employee, as one participant said:

‘*I have actually been confirmed through this work we have done here, that it [senses and emotions] has value, and you can listen to the other dimensions [senses and emotions]. They actually tell you much more than you can rationalize your way to, and that you can navigate much more after them, and I actually see them as part of my own individual capital.’*

### 4.3 Learning to witness oneself and surroundings

As participants embraced their emotions unconditionally, they experienced that using their senses as the internal navigator helped them become more present and better observe their surroundings. Participants began to train the MER Inquiry Practice outside of the therapeutic training sessions and slowly increased their ability to observe and become aware of themselves and surroundings in various contexts. Participants had previously been occupied with feelings of not being good enough and a want to rationally figure everything out before it happened. Training the MER Inquiry Practice increased their ability to slowly calm themselves more through focusing on their senses, taking deep breaths and being more open to uncomfortable emotions. Through continued training, they became increasingly aware of their previously unconscious patterns and were no longer reacting automatically as often as beforehand, as one participant expressed:

*‘Before I reacted completely automatically and things kept being the same, it was the same pattern I repeated and repeated. [Now] I breathe, then I relax, and I think to myself, what is this about? What do I sense? And [I] observe the emotions that arise in me. Before, I would have defended myself or scolded or started a discussion or held on to mine, where [now] I observe a bit and say, some people react, they do something that I do not like, but is it theirs or is it mine … I have become better at standing still and staying calm while still being able to sense.’*

This ability to gain insights into the present through sensory awareness enabled participants to sense the energy and emotions coming from other people beyond their words. Participants became increasingly better at noticing when others expressed one thing but meant another, as one participant said: *‘I have opened my eyes for something that is being said but not meant and that there is a mistrust in our organization in some places and it is really unhealthy’.* When engaging with mistrustful and destructive cultures participants at times felt uncomfortable and insecure, being pulled back into their old patterns of overthinking and suppressing emotions, as one participant expressed:

*’A pull in the stomach*. *An uncertainty of how it [sharing feelings and opinions] will be received. You can bring something pure, but if the reaction or answer you get is not pure, then it is hard to sense [due to] a baggage from home where you say it's fine, even if it's not fine.’*

However, these experiences also helped participants to become more aware of their inner patterns, helping them to dismantle their defensive mechanisms, as one participant expressed:

*'Now I have repeated the concept [MER Inquiry Practice] so many times and now it makes sense with thoughts and emotions and the mindset and this concept of sensing through the body and having a focus on sensing, because it tells me what is happening … For me, it has been about dismantling these weapons and learning how I function, and that has simply been worth gold to learn to get behind my own defensive mechanisms.'*

### 4.4 Learning to interact openly with others

The more participants observed their surroundings, the more aware they became of the kind of culture they wanted to facilitate and be a part of. Participants found that acting more and more through their body sensations created a more honest and open environment and enabled them to influence people and culture both within the focus group and outside of it. Through continued training, participants became increasingly skilled at picking up underlying intentions and feelings in others and thereafter adapt appropriately to increase collaboration and social capital, as one participant said:

*‘The sharpening of my sensory apparatus in this cultural project means that I can read those artifacts in a split second, people do not have to open their mouths much before you are aware of what you are facing or [you] have an artifact, where you yourself reflectively [automatically] shut down and say this is uncomfortable. My experience tells me that if you go in and grab a hold of it, stretch your hand out and start working into the field, then you will be able to move that artifact and bring it out to something where it makes sense, where you can get experience that can still end up being positive. I have to pull myself together, clench the teeth and then work into the field. I have experienced that it can succeed, and it always succeeds and comes out at the other end being positive.'*

Participants started slowly taking more responsibility of directly facilitating more collaboration and trust with their surrounding employees, actively taking on the responsibilities as change agents for a better culture, as one participant expressed:

*'The [focus] group we have formed, there we have opened up to a very high degree of sincerity and honesty, which is very, very strong, and it ends up being in contrast with the normal collegial atmosphere where there are some things we talk about and some things we do not talk about, and where we as culture-carriers [change agents] or I as a culture-carrier [change agent] am training to bring my new skills into the space with my colleagues … If I change, I also force my surroundings to change, because I am no longer the same, they no longer play ball up the same wall if my wall suddenly looks different and that is really exciting.'*

Participants began to share and initiate conversations about more emotionally and practically difficult things to increase collaboration and to better take on difficult work tasks and situations, as one participant expressed:

*'It feels nice, comfortable, this sense of energy and desire for changeability, a desire for more. I experience that we share things that are significantly more difficult than what we have done before, it can be both professionally difficult tasks, but it can also be things on the personal level. There are still room for improvement, but we are definitely on the way.'*

Participants found these active changes in their behaviors to increase social capital across relationships, and participants began to experience a more un-politicized and frictionless culture, creating higher quality of work and better products.

### 4.5 Learning to continuously practice Safe Harbor

At a FSC group meeting during phase 3 of the study, the concept of *Safe Harbor* was developed. Safe Harbor is the practice of continuously developing oneself and the surrounding culture by continuously training one's sensing abilities and approaching emotional resistance and challenges with curiosity and openness. Practicing Safe Harbor means that an individual has become so trained in their sensory and emotional abilities that they can constantly bring themselves to an ‘unfrozen’ stage as needed to adapt and develop themselves for the betterment of the overall culture and FSC. This became the goal for both the FSC group and focus group as they wanted to facilitate a continuously developing culture of trust and increase social capital in the department and with various partners. One participant expressed the practice of Safe Harbor as loving, truthful, and a win-win for all parts:

*‘It is the love to another human being that drives this [practice], and if that is what we put on the table, our love and truth. They can sense it and that way you get this synergy with another human being. It has such a huge impact, it is un-politicizing, and it is a win-win, and that means a lot to me that I can sense that there is no one that needs to win here. There is not one opinion that has to get in front of another’s opinion, because we do have a lot in our culture, where we have to brag and knock others in place, because ‘I want my opinion’. That is not the case with this wonderful way of working, instead you are explorative, explorative together, and arrive together to a result where everyone can agree, it was a win-win, we are together in this.'*

Safe Harbor always starts in oneself before you can bring it out in relations with others, which one participant described as reaching a balanced state, where emotions were allowed to exist freely within themself, and their mind was open and focused on sensory perceptions. Another participant described this state as a balance between feeling calm and empowered. The more balanced they became within themselves, the more they became able to change how they interacted with others, as the participant expressed:

‘*I obviously have a huge calmness and at the other end of the pendulum, I clearly have a huge, huge force. In the calmness exists also the feeling of safety and the laid back-ness that can make me tired. At the other end is the energy and the avidness, the impatience and there is no doubt that there is a balance somewhere in the middle, a dynamic dose of things, gives me this feeling of harmony or balance, and it can make me experience that I am in inner synergy with myself. I clearly have a multidimensional dynamic between my inner pendulum and a pendulum from the inside and out in reality. There are two pendulums you can say, an inner dynamic pendulum swinging that when it swings and lands then it actually allows the other one to move, very liberating, very calming.’*

### 4.6 Change Agent Model for culture development

The participants identified Safe Harbor as the best practice to strive for to become culture change agents and lead the continuous development of the cultures and FSCs around them. Learning to continuously practice Safe Harbor was hard work, as participants had to continuously go through the other four underlying mechanisms: Accepting senses as the internal navigator, embracing one's emotions unconditionally, witnessing oneself and surroundings, and interacting openly with others, as illustrated in the Change Agent Model, Model 5. To become a culture change agent, an individual must be willing to take responsibility to continuously move through the different underlying mechanisms in the model, dealing with what is difficult for oneself, to continuously bring oneself in Safe Harbor and facilitate others to do the same.

**Model 5:**
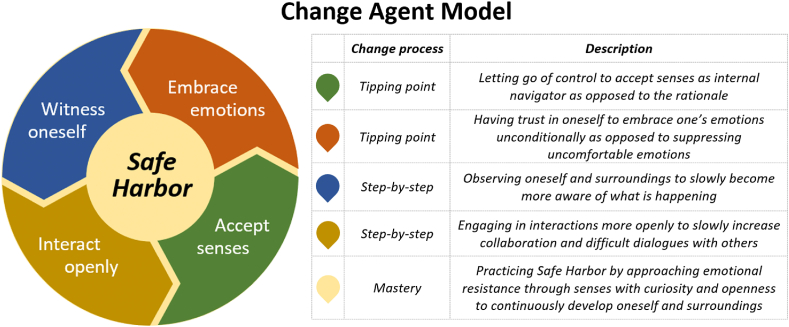
**Change Agent Model:** The model illustrates the continuous loop that individuals must be willing to engage in to create Safe Harbor in themselves and with others, and thereby transform their cultural essence and the cultures and FSCs around them. All underlying mechanisms are described next to the model.

## 5 Discussion

### 5.1 Developing individuals who are ready and willing

The study suggests that the transformational journey of addressing underlying mechanisms of oneself and the culture, should be engaged in voluntarily and on whatever level an individual feels ready for themselves, as enforcing practices may lead to more emotional resistance to change [25]. This is especially important for this type of culture development as addressing underlying mechanisms through therapeutic training undoubtedly will have consequences for all aspects of the individual's life. Individuals must be both ready and willing to develop themselves and face aspects of themselves that may previously have been unconscious to them. The study also highlights the importance of taking on the challenge of becoming culture change agents as a group to allow for a safe space where everyone is committed to exploration and experimentation. Such an environment resembles psychological safety, where people can comfortably be themselves, share concerns and mistakes openly, and engage in new experimental practices without fear of negative consequences of self-image, status, or career [26,41]. This study therefore recommends the identification of individuals with a high level of readiness, and the investment of significant resources and support for them to develop their abilities as culture change agents.

### 5.2 Training human competencies to develop sensory and emotional skills

The study showed that individual's capacity to positively influence culture is limited by their ability to positively develop themselves. To become a change agent for FSC and organizational culture development, an individual should begin by looking inward and training their human competencies. Specifically, individuals should work towards accepting senses as their internal navigator as opposed to the rationale. Other researchers have found that individuals often interpret the world through rationally created frames of references known as ‘mental maps’ [25] or ‘self-referentiality’ [42] that inhibit individuals by creating fixed ways of reacting and engaging in different situations. To overcome frames of references, individuals should actively train to bring what they sense to awareness and learn to distinguish it from what they feel emotionally. This allows for a more present-centered and emotionally open approach to oneself and surroundings. The emotional opening can then be used to work towards embracing all one's emotions unconditionally and truly recognize all emotions as valuable and useful. For some people, it can be expected to take therapeutic training to work through one's emotions. This is normal, as most people are never taught how to healthily deal with their emotions. Learning to embrace one's emotions unconditionally is essential for culture and FSC development, as emotional suppression may otherwise cause individuals to unconsciously project their uncomfortable feelings onto the situation they are in, causing destructive behavior for the group and culture [43].

### 5.3 Observing and acting differently for a better FSC and organizational culture

Training one's sensory and emotional skills is an important foundation to work towards more accurately witnessing oneself and surroundings, and to learn to interact more openly to solve complex and critical challenges. Becoming better at witnessing oneself and surroundings by focusing on senses and emotions may increase an individual's ability to notice disparities between talk and action, helping them to get to the root cause of issues they face. This is an important step in training individuals to ‘raise the hand’ despite emotional resistance, which is crucial to develop FSC [44]. Learning to interact more openly and increase social capital by bringing one's internal emotional trust into relationships may facilitate more sharing of learned experiences and new learning from near-misses and failures, and a higher ability to take action when faced with uncomfortable situations or conflicts. Several studies according to a literature review and current regulations related to FSC have highlighted the importance of all these abilities in developing FSC [1,9,18].

### 5.4 Safe Harbor as a continuous practice for culture change

To become a culture change agent, the study suggests that individuals should work towards practicing Safe Harbor within themselves and with others. Safe Harbor reflects the practice of approaching emotional resistance through senses with curiosity and openness, thereby actively using one's sensory and emotional abilities to develop oneself and influence the culture and FSC. Continuing to engage in all underlying mechanisms as needed to create Safe Harbor is essential to ensure ongoing development, as illustrated in the Change Agent Model, see Model 5. This reflects ‘generative learning’ or ‘a continuous loop of learning’, where individuals become able to learn to learn and thereby ongoingly take on increasingly interdisciplinary challenges and challenge former solutions [26,27]. Being able to practice Safe Harbor enables an individual to continuously develop and thereby bring themselves in an ‘unfrozen’ stage as needed. This ability reflects a general culture or FSC that actively leans into working through crises to better themselves and the safety of their products as opposed to ignoring problems and doing everything possible to keep the status quo. In the context of FSC, there is a need to facilitate a more wide-spread engagement in transforming FSC at its root causes as opposed to only dealing with food safety crises at the superficial layer [18]. Being able to work through food safety challenges is needed in any FSC, no matter the pressure on production orders or disparity between talk and action in local managers [19]. Creating culture change agents capable of practicing Safe Harbor may therefore be relevant in any context where individuals or organizations seek to create a more honest, trustful, and collaborative culture and FSC.

## 6 Study limitations and novel methods

The study was conducted using a qualitative and explorative approach going in-depth with few participants to reveal underlying mechanisms. The study specifically explored underlying mechanisms connecting transformation of individuals’ internal states with change to the culture and social capital of the department and overall organization. The therapeutic training (action intervention) worked deeply with voluntary participants over a long period of time, making it too time-consuming and/or emotionally challenging for some employees. This was done to prioritize reaching deeply enough into the underlying cultural layers with each volunteer to make them agents of change in the department. The subjective experiences of a limited number of participants may not be directly generalizable to others as they reflect their specific lived experiences. The study overcame this limitation by working deeply with each participant to access the underlying mechanisms of their individual culture, which has implications for any culture and FSC development process. This is due to the inherent nature of underlying assumptions in culture, as the individual human process of bringing underlying mechanisms to awareness remains similar for any individual, no matter the specific culture they seek to develop. Furthermore, the study developed a new question framework for in-depth interviews through which participants were facilitated to access their underlying assumptions by going through the trained MER Inquiry Practice. This allowed for the deeper access to underlying mechanisms to also be reflected in the data. When identifying underlying mechanisms, the study added the categorization of change processes to clarify the individual transformation processes connected to the different underlying mechanisms. The underlying mechanisms identified may serve as a starting point for further studies, exploring the individual transformation process of changing culture through action research.

## 7 Conclusion

The study showed that working intensely and therapeutically with senses and emotions on an individual and group level can have a significant impact on the surrounding culture and social capital by bringing underlying mechanisms to awareness. The study revealed five underlying mechanisms that linked individual culture change to the social capital and culture development in the department. The five underlying mechanisms identified were: Accepting senses as the internal navigator, embracing one's emotions unconditionally, witnessing oneself and surroundings, interacting openly with others, and practicing Safe Harbor. These underlying mechanisms are relevant for any culture, including FSC, that seek to create long-term behavior and culture change. Collectively, the underlying mechanisms make up the Change Agent Model, see Model 5. The model reveals important underlying mechanisms to create Safe Harbor that any individual or group can work through to become culture change agents and drivers for organizational culture and FSC development.

This is the first of two articles. The second article will explore human development typologies that were created during focus group meetings to conceptualize different approaches to human learning. The second article will also go more in depth with the practice of bringing senses and emotions to awareness and discuss its application for leadership development and scientific inquiry.

## Author contribution statement

Anne-Mette Olsen: Conceived and designed the experiments; Performed the experiments; Analyzed and interpreted the data; Contributed reagents, materials, analysis tools or data; Wrote the paper.

Anna Marie Møller: Conceived and designed the experiments; Analyzed and interpreted the data; Contributed reagents, materials, analysis tools or data; Wrote the paper.

Sanne Lehmann: Conceived and designed the experiments; Contributed reagents, materials, analysis tools or data; Wrote the paper.

Anders Vind Kiethon: Conceived and designed the experiments; Performed the experiments; Contributed reagents, materials, analysis tools or data; Wrote the paper.

## Funding statement

This work was supported by the Danish Agriculture and Food Council.

## Data availability statement

The data that has been used is confidential.

## Declaration of interest's statement

The authors declare no conflict of interest.
